# Swill and Pig Manure Substrates Differentially Affected Transcriptome and Metabolome of the Black Soldier Fly Larvae

**DOI:** 10.3390/ijms252212147

**Published:** 2024-11-12

**Authors:** Bin Zhang, Rencan Yang, Shichun He, Sifan Dai, Qingquan Hu, Xinrong Li, Hongren Su, Jingyi Shi, Zhiyong Zhao, Dongwang Wu

**Affiliations:** 1 Yunnan Academy of Animal Husbandry and Veterinary Sciences, Kunming 650224, China; binzhang89@163.com (B.Z.); yangrenchan@126.com (R.Y.); swau023@163.com (Q.H.); lxrkm2000@126.com (X.L.); 2 Yunnan Provincial Key Laboratory of Animal Nutrition and Feed, Faculty of Animal Science and Technology, Yunnan Agricultural University, Kunming 650201, China; heshichun0529@163.com (S.H.); 15987179618@163.com (S.D.); shr2904317687@163.com (H.S.); shijingyi3@163.com (J.S.)

**Keywords:** black soldier fly (*Hermetia illucens*), transcriptomics, metabolomics, substrate effects, gene expression

## Abstract

Black soldier fly larvae (*Hermetia illucens*) (BSFL) are insect larvae with significant ecological and economic value. This study aims to investigate whether swill and manure had any effects on the transcriptome and metabolome of BSFL. Through high-throughput transcriptome sequencing, we found that larvae fed with swill exhibited higher levels of gene expression, especially with the upregulation of genes related to energy metabolism, amino acid metabolism, and redox reactions. Metabolomics analysis showed a significant increase in energy metabolism-related metabolites, such as organic acids and amino acids, in the swill-fed larvae. In contrast, gene expression and metabolic characteristics in the pig manure-fed group indicated a higher stress response, with relevant genes and metabolites (such as short-chain fatty acids and antioxidants) showing significant upregulation. GO and KEGG enrichment analyses further supported these results, suggesting that swill promotes larval growth and metabolism, whereas pig manure induces the activation of stress response mechanisms. These findings offer clear molecular and physiological insights into the optimization of substrate selection for enhancing the performance of BSFL in waste management.

## 1. Introduction

The black soldier fly (*Hermetia illucens*) is an insect species with a global distribution, which attracts significant attention for its potential in organic waste treatment and resource recycling [1,2]. Its larvae are capable of converting organic waste (such as swill, animal manure, and agricultural waste) into valuable biomass, including proteins, lipids, and other useful compounds, which can be applied in industries like energy, food, feed, and agriculture [3,4,5]. This highly efficient waste conversion ability makes the black soldier fly a crucial component of environmentally friendly bioprocessing technologies [6]. The larvae of the black soldier fly can grow and develop on a variety of organic substrates, significantly reducing waste volume within a short time while transforming it into economically valuable nutrients suitable for the production of animal feed and biofertilizer [7,8]. In the context of global climate change and the depletion of natural resources, meeting the growing demand for animal feed and human food with sufficient, nutritious, safe, and affordable protein sources has become an urgent challenge [9,10]. Additionally, black soldier fly larvae have demonstrated notable bioremediation capabilities, including resistance to antibiotics and heavy metal pollutants [11]. As a result, BSFL hold significant potential as a sustainable resource for addressing global environmental and nutritional challenges [12]. Black soldier fly larvae, with their ability to convert organic waste into high-value biomass rich in proteins and lipids, offer a promising solution to the global need for sustainable protein production, while simultaneously addressing waste reduction.

Although the black soldier fly’s applications in waste management are well established [13], the biological mechanisms by which different organic waste substrates influence its growth, development, and metabolic activity remain underexplored. Previous research has mainly focused on the growth rate, biomass yield, and nutrient composition of larvae fed on various substrates, as well as their ability to degrade antibiotics and pathogens [14,15,16]. However, there is a paucity of studies investigating the gene expression and metabolic responses of larvae to divergent substrates at the molecular level, particularly using advanced omics approaches. Swill and pig manure are among the most commonly produced organic waste types in practical waste management, with swill generated at an estimated rate of 200 to 300 tons daily in larger urban areas, and pig manure at approximately 100 to 200 tons daily in large-scale pig farming operations. Each possesses distinct biological characteristics [17,18]. Swill, which is composed of proteins, fats, and carbohydrates, is highly variable and complex due to its diverse sources, whereas pig manure is rich in nitrogen and minerals, enhancing its role as a fertilizer component [19]. These compositional differences between swill and pig manure are likely to exert differential impacts on the physiology and metabolism of black soldier fly larvae. Given that both substrates are produced in enormous quantities daily, understanding their effects on larvae at the molecular level is essential for optimizing waste management strategies. Despite the established applications of the black soldier fly in waste management, the physiological mechanisms driving its metabolic responses to different substrates remain largely unexplored. Understanding how the nutritional composition and environmental factors of these substrates influence larval gene expression and metabolism is critical for enhancing their efficiency in waste reduction and nutrient conversion. How do different substrates (swill and pig manure) affect the gene expression and metabolism of BSFL? Do substrates with distinct nutrient compositions (swill vs. pig manure) induce different physiological or stress responses in BSFL? We hypothesize that swill, with its higher nutritional diversity, will promote growth and metabolism in BSFL, while pig manure will trigger a greater stress response due to its high nitrogen and mineral content. This research aims to fill this knowledge gap by investigating the molecular responses of black soldier fly larvae to two contrasting waste substrates, providing insights into how these substrates influence key physiological processes regulating growth, development, and stress responses.

Additionally, we acknowledge the high complexity and variability of swill compared to pig manure, and that results may differ with different types of swill. This variability emphasizes the importance of our study, as understanding these differences at the molecular level can inform better management practices and enhance the performance of BSFL in diverse waste environments. This study contributes valuable data to ongoing efforts to enhance the performance of black soldier fly larvae in waste reduction and resource recycling, providing scientific evidence for improving substrate utilization in large-scale applications.

## 2. Results

### 2.1. Overall RNA-Seq Results and Quality Control

To investigate the effects of swill and pig manure treatment on *Hermetia illucens* larvae, a total of approximately 757,945,542 high-quality reads were obtained through transcriptome sequencing (with an average of about 63,162,128 reads per sample). The Q30 score of the samples was greater than 94%, and the efficiency of the alignment with the reference genome was greater than 80%. Approximately 30,000 genes were expressed in at least one sample. Based on the expression level (FPKM), the number of genes in each sample with different expression abundances was calculated (FPKM ≥ 50, 0 ≤ FPKM < 1, 1 ≤ FPKM < 5, 5 ≤ FPKM < 10, 10 ≤ FPKM < 30, 30 ≤ FPKM < 50, 50 ≤ FPKM; Supplementary Table S1). 

### 2.2. Differential Gene Expression Analysis

**Figure 1:**
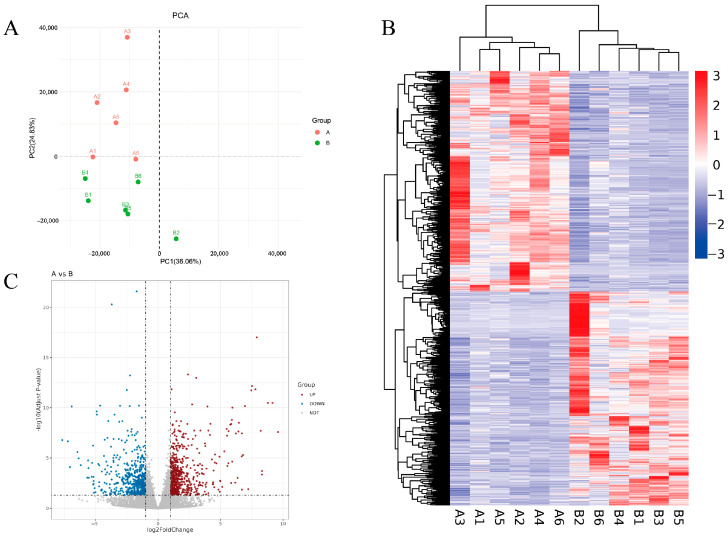
(**A**) Two-dimensional Principal Component Analysis (PCA) plot. (**B**) Differential gene clustering heatmap. Note: Each column represents a sample and each row represents a gene. Red indicates relatively high expression of genes, while blue indicates relatively low expression of genes. The dendrogram above represents the clustering of the samples, where the closer two branches are, the more similar the expression patterns of the differential genes in those samples. The dendrogram on the left represents the clustering of the genes, where the closer two gene branches are, the more similar their expression levels. (**C**) Volcano plot of differentially expressed gene distribution. Note: The horizontal axis represents the fold change in gene expression between different experimental groups or samples, and the vertical axis represents the statistical significance of gene expression changes. Each dot represents a gene, with gray dots indicating non-significantly different genes, red dots indicating significantly upregulated genes, and blue dots indicating significantly downregulated genes.

To analyze the dynamic transcriptomic changes under the stress of two substrates, PCA (Principal Component Analysis) was performed based on the gene expression data from all samples. The distances between the two groups of samples represented the overall intergroup expression differences. Notably, samples A3 and B2 were farther apart, indicating a larger transcriptomic difference between these two individuals (Figure 1A).

Cluster analysis of genes was conducted based on the changes in differential gene expression between groups and the similarity of gene expression in the samples (Figure 1B). The heatmap of differentially expressed genes showed that *Hermetia illucens* larvae exhibited similar expression levels in response to both swill and pig manure treatments. However, larvae treated with swill had more upregulated differentially expressed genes, while larvae treated with pig manure exhibited more downregulated differentially expressed genes compared to the swill group. A volcano plot revealed the number of upregulated, downregulated, and unchanged genes (Figure 1C).

### 2.3. GO and KEGG Enrichment Analysis

**Figure 2:**
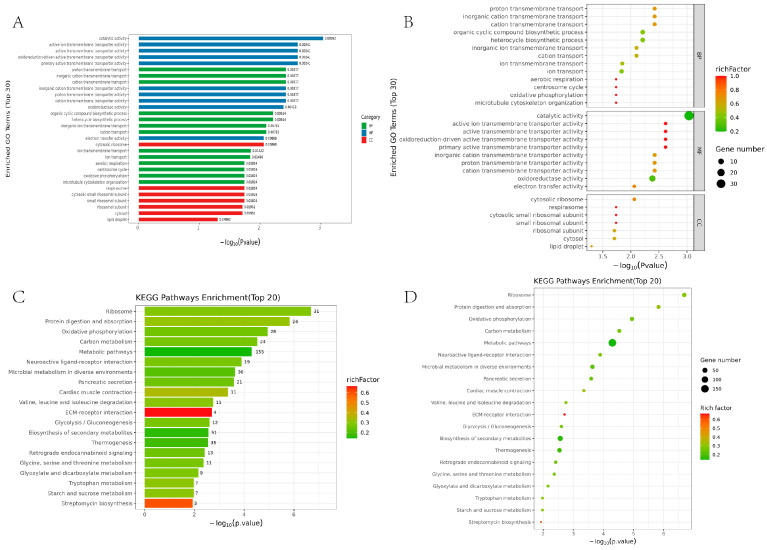
(**A**) Bar chart of GO enrichment for differentially expressed genes. The horizontal axis represents −log10 of the adjusted *p*-value, with entries arranged in ascending order of *p*-value. The three main categories of GO (green for biological processes, blue for molecular functions, and red for cellular components) are represented by different colored bars, while the vertical axis describes specific functions. (**B**) Bubble chart of GO enrichment for differentially expressed genes. The horizontal axis represents −log10 of the adjusted *p*-value, with entries arranged in ascending order of *p*-value. The three main categories of GO are shown from top to bottom: biological processes (BPs), molecular functions (MFs), and cellular components (CCs), while the vertical axis provides descriptions of specific functions. (**C**) Bar chart of KEGG enrichment for differentially expressed genes. The horizontal axis represents −log10 of the adjusted *p*-value, while the vertical axis displays pathway names. The size of the rich factor is indicated by the color of the bars, and the number of differentially expressed genes within each pathway is shown on the right side of the bars. (**D**) Bubble chart of KEGG enrichment for differentially expressed genes. The horizontal axis represents −log10 of the adjusted *p*-value, while the vertical axis shows pathway names. The size of the rich factor is indicated by the color of the bubbles, with larger values indicated by colors closer to red. The size of the bubbles represents the number of differentially expressed genes in each pathway.

To explore the effects of different substrates on the metabolic pathways of *Hermetia illucens* larvae, we conducted GO enrichment analysis on the differential genes. The GO annotation analysis revealed a significant number of differentially expressed genes involved in biological processes, followed by molecular functions, while fewer genes were involved in cellular components. The differential genes were associated with 13 biological processes, 10 molecular functions, and 7 cellular components (Figure 2A). The biological processes with the highest number of differential genes were catalytic activity, oxidoreductase activity, and cellular nitrogen compound biosynthetic process (Figure 2B).

The KEGG enrichment analysis found that 34 KEGG pathways were significantly enriched in the *Hermetia illucens* larvae treated with swill and pig manure. The top 20 significantly enriched pathways primarily involved metabolic pathways, biosynthesis of secondary metabolites, thermogenesis, and microbial metabolism in diverse environments (Figure 2C). We further investigated the pathways related to carbon metabolism (ko01200), metabolic pathways (ko01100), microbial metabolism in diverse environments (ko01120), biosynthesis of secondary metabolites (ko01110), glycine, serine and threonine metabolism (ko00260), glyoxylate and dicarboxylate metabolism (ko00630), tryptophan metabolism (ko00380), and starch and sucrose metabolism (ko00500). The results indicated that swill and pig manure substrates significantly promoted the metabolic activity of *Hermetia illucens*. Among the top 20 significantly enriched KEGG pathways, 8 pathways were involved in metabolic processes (Figure 2D).

### 2.4. Metabolomics Analysis

**Figure 3:**
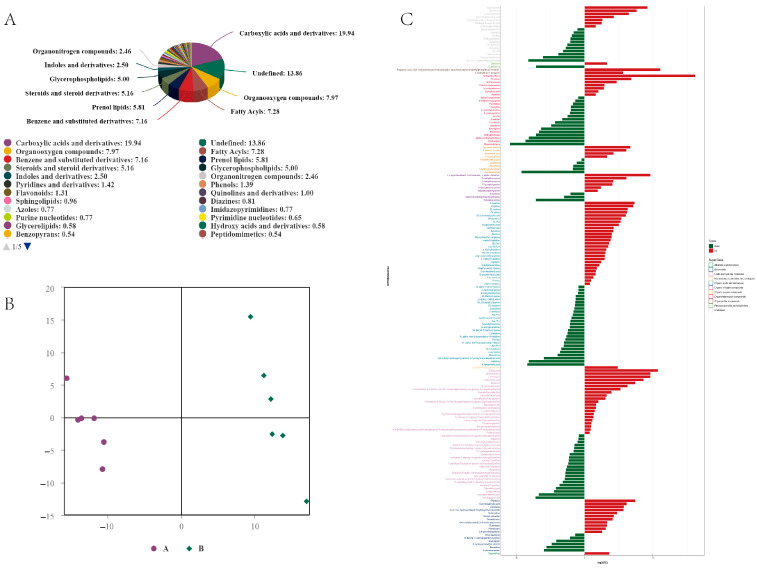
(**A**) The proportion of identified metabolites in each chemical classification. Note: The different colored blocks in the figure represent various chemical classification categories, and the percentage indicates the proportion of metabolites within that chemical classification relative to the total number of identified metabolites. Metabolites without a defined chemical classification are categorized as undefined. (**B**) OPLS-DA score plot for positive ion mode. Note: The *x*-axis [1] represents principal component 1, while the *y*-axis [2] represents principal component 2. Points of the same color represent biological replicates within the same group, and their distribution reflects the differences between and within groups. (**C**) Analysis of the fold change in expression of significantly different metabolites in positive ion mode. Note: The *x*-axis indicates the log2 FC values of the differential metabolites, which represent the fold change in these metabolites on a log2 scale. The *y*-axis represents the significantly different metabolites. Red indicates upregulated differential metabolites, while green indicates downregulated differential metabolites.

**Figure 4:**
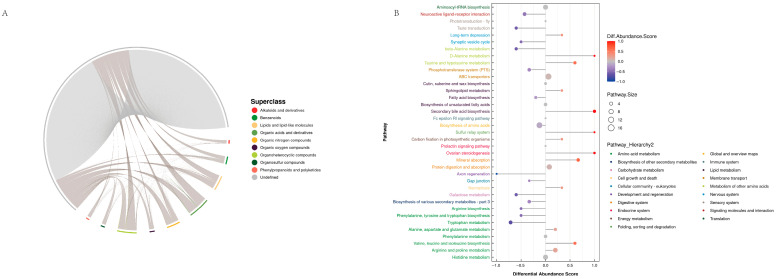
(**A**) Chord diagram in positive ion mode. Note: The inner circle links represent significant differential metabolites, while the arcs on the outer circle indicate the categories of these metabolites. The colored lines represent correlations within each metabolite category, with lines matching the color of their respective subclasses. Dark gray lines represent correlations between different categories of metabolites. (**B**) Differential abundance score plot for all differential metabolic pathways. Note: The Y-axis represents the names of the differential pathways, while the X-axis shows the differential abundance score (DA score). The DA score reflects the overall change in all metabolites within a metabolic pathway. A score of 1 indicates an upregulation trend for all identified metabolites in that pathway, while 1 indicates a downregulation trend. The length of the line segment represents the absolute value of the DA score, and the size of the endpoint circles indicates the number of metabolites within that pathway—larger circles signify more metabolites. The color intensity of the lines and circles corresponds to the DA score value, with deeper red indicating a stronger upregulation trend and deeper blue indicating a stronger downregulation trend.

In this study, a total of 2598 metabolites were detected, including carboxylic acids and derivatives (19.94%), organooxygen compounds (7.97%), fatty acyls (7.28%), and benzene and substituted derivatives (7.16%), with undefined compounds making up 13.86%. Other substances accounted for less than 5% (Figure 3A). Partial Least Squares Discrimination Analysis (OPLS-DA) was employed to distinguish the differences between the comparison groups, revealing significant differences between the two groups (Figure 3B). Further analysis using OPLS-DA and *p*-values for screening differential metabolites (criteria: OPLS-DA VIP > 1 and *p*-value < 0.05) identified a total of 180 differential metabolites in the comparison of groups A and B, with 91 metabolites being upregulated and 89 metabolites downregulated (Figure 3C). To intuitively illustrate the co-regulatory relationships among different metabolites, a chord diagram was used to show pairs of metabolites with a correlation coefficient |r| > 0.5 and *p* < 0.05. Organic acids and derivatives, organic oxygen compounds, and organoheterocyclic compounds exhibited high co-correlation (Figure 4A). Differential metabolites from the different comparison groups were matched against the KEGG database to obtain pathway information involving these metabolites. Enrichment analysis of the annotated results revealed that the differential metabolites in groups A and B were mainly annotated and enriched in the pathways of protein digestion and absorption, ABC transporters, and biosynthesis of amino acids (Figure 4B).

## 3. Discussion

Currently, studies on the effects of different substrates on gene expression and metabolism in black soldier fly larvae (BSFL) are relatively limited. Most existing research focuses more on their growth performance, nutritional composition, and application in organic waste treatment, with few reports on gene expression and metabolic pathways. This type of study could provide more biological insights into the adaptive mechanisms of BSFL under different substrate conditions and help optimize their application in industrial production. This study combines transcriptomic and metabolomic analyses to explore the physiological and metabolic effects of swill and pig manure substrates on BSFL. By comparing our results with previous studies, our research further elucidates the impact of different organic waste substrates on the metabolic regulation mechanisms of BSFL.

As a high-value insect, BSFL have been extensively studied by researchers considering a range of applications, such as their ability to degrade heavy metals and produce chitosan and antimicrobial peptides [20,21,22]. BSFL can efficiently degrade organic waste and convert it into fat-rich insect biomass, making them widely applicable in waste management and feed production [23]. Zhu et al. revealed the molecular mechanisms of fat accumulation in BSFL through transcriptomic analysis [24]. Swill and pig manure, as different substrates, significantly affected the metabolic pathways of BSFL. Our study found that BSFL in the swill-treated group exhibited higher gene expression levels, particularly with regard to genes related to energy metabolism, amino acid metabolism, and redox reactions, which were significantly upregulated [25]. Similarly, Xu et al. indicated that substrate changes can influence gene expression in BSFL, which is consistent with our findings [26]. In particular, genes related to energy metabolism, such as those involved in the tricarboxylic acid cycle, glycolysis, and fatty acid oxidation, were significantly upregulated in the swill substrate group, suggesting that the protein- and lipid-rich composition of the swill provided ample nutrients for larval growth [27]. Other studies have found that feed composition significantly affects the growth efficiency of BSFL and their generation of metabolic products, which aligns with our results [28]. In contrast, BSFL in the pig manure-treated group exhibited upregulation of genes related to stress response and antioxidant defense [29]. This may be due to the presence of a large microbial community and their metabolites, such as short-chain fatty acids and ammonia compounds, in pig manure, which might impose physiological stress on the larvae. Some studies have noted that the complex microbial community and potential toxic substances in pig manure substrates may induce stress responses in larvae [30,31].

GO and KEGG enrichment analyses further verified the differential effects of these substrates on gene expression. Notably, the differentially expressed genes in the swill-treated group were enriched in metabolic pathways such as carbon metabolism, amino acid synthesis, and secondary metabolite synthesis, while the genes in the pig manure-treated group were enriched in pathways related to antimicrobial peptide production and immune defense [32,33]. These findings suggest that BSFL can adapt to different substrate nutrient environments by regulating gene expression, thereby optimizing their growth and survival strategies [34]. The metabolomics analysis results further supported the findings of the transcriptomics analysis. In the swill substrate, BSFL exhibited higher levels of metabolites such as organic acids, amino acids, and triglycerides, which are important intermediates in energy metabolism, indicating that the larvae experienced higher metabolic activity in this substrate [18]. In contrast, higher levels of stress-related metabolites, such as short-chain fatty acids and antioxidants, were detected in the pig manure-treated group, consistent with the physiological stress response of the larvae in this substrate.

In summary, this study, through integrated transcriptomic and metabolomic analysis, reveals how BSFL adapt to different substrate environments by regulating metabolic pathways. It also discusses a range of roles played by BSFL, including the following: Ecological Role: BSFL, which show enhanced energy metabolism, could contribute more effectively to organic waste recycling by converting diverse waste into valuable biomass. This would align with the role BSFL play in bioremediation and sustainable waste management. Industrial Role: The findings of enhanced metabolism and growth in BSFL can be used to optimize waste conversion strategies in large-scale industrial applications (e.g., animal feed production). In contrast, the stress responses in BSFL could inform substrate optimization practices, improving efficiency in environments where manure is a dominant waste type. This finding not only helps us to understand the ecological adaptation strategies of BSFL in organic waste degradation but also provides a theoretical basis for optimizing their application in waste management. Adjusting and optimizing substrate composition will help improve the growth efficiency of BSFL and their waste conversion rate, thereby enhancing the economic benefits and sustainability of resource recycling. Focusing more on the metabolic performance of BSFL during growth will help clarify their role in complex environments [35,36].

## 4. Materials and Methods

**Figure 5:**
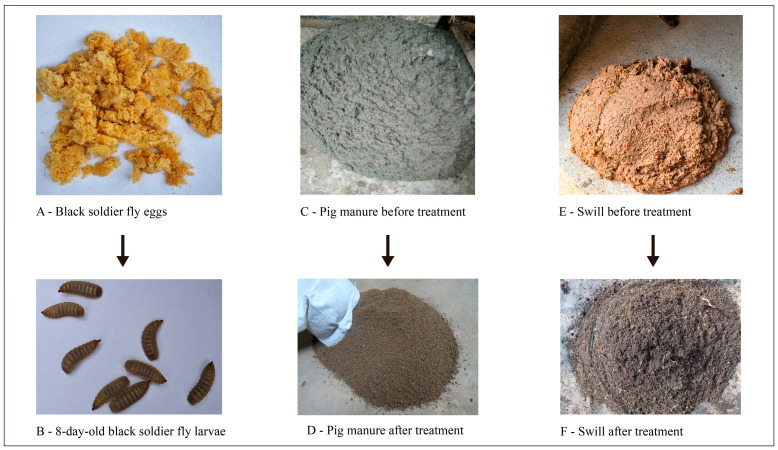
Swill and pig manure substrates significantly influenced the transcriptome and metabolome of black soldier fly larvae, with the figure showing specific samples before and after treatment at each stage.

The black soldier fly larvae used in this experiment were sourced from two groups: Group A was obtained from the Yunnan Provincial Institute of Animal Science and Veterinary Medicine and was primarily used for processing restaurant swill from Kunming City. Group B consisted of a population isolated from Group A in 2018, which was primarily used for processing pig manure. Both Group A and B larvae were tested for rearing density, with larvae placed in 4 m² treatment pools to process swill and pig manure. The nutritional components are shown in Table 1. The initial density of larvae was 250,000 per m² (5-day-old larvae). The design of the experiment and effect of the treatment are shown in Figure 5.

Treatment process: At the beginning of the experiment, a certain amount of swill (or pig manure) was placed into the treatment pools, followed by the introduction of 5-day-old black soldier fly larvae. Once the swill or pig manure was processed into a sand-like texture, additional swill or pig manure was added depending on the processing time. The swill used in the experiment was collected from urban restaurants in Kunming, after undergoing three-phase separation (i.e., removal of water and oil). The remaining solid residue was further ground into a slurry, with a moisture content between 77% and 85%. After being processed by the black soldier fly larvae, the frass (larval waste) became visually distinguishable, with a layer of dry, non-processable materials like chili skins or fibrous matter floating on the surface. The frass appeared granular and dark brown, signaling the completion of the treatment. At this point, more swill was added. The moisture content of the frass after swill treatment was between 45% and 50%.

The pig manure was collected from pigpens that primarily used dry-clean manure systems. It was mixed evenly using a mixer, with a moisture content between 75% and 80%. After processing by the black soldier fly larvae, the frass showed no signs of clumping, indicating the completion of treatment. The moisture content of the frass after pig manure treatment was between 50% and 55%. The changes in the body weight of black soldier fly larvae at different stages are listed in Table 2.

All samples used were from the larval stage of the black soldier fly, with the larvae appearing white and not yet in the pre-pupal stage (where the body turns black). Samples of larvae from the swill and pig manure groups were collected on the eighth day after being placed in the treatment pools, with individual larvae weighing between 0.18 g and 0.25 g. Sixty black soldier fly larvae were randomly selected from each group, immediately euthanized using chloroform, and repeatedly washed with sterile water. The larvae were then dissected using sterile dissection forceps to separate different tissues (cuticle, fat body, gut, Malpighian tubules, and hemolymph). The tissues from 10 larvae were pooled to form a single sample, which was promptly transferred into liquid nitrogen for storage, to be used for RNA and metabolomic analysis.

### 4.1. Experimental Methods

**RNA Extraction and Sequencing:**

Total RNA was extracted from black soldier fly larvae using TRIzol^®^ reagent (Magen, Beijing, China). The A260/A280 absorbance ratio of RNA samples was measured using a Nanodrop ND-2000 (Thermo Scientific, Waltham, MA, USA), and the RNA integrity number (RIN) was determined using an Agilent Bioanalyzer 4150 (Agilent Technologies, Santa Clara, CA, USA). RNA samples that passed quality control were used for library construction. A paired-end (PE) library was prepared according to the instructions of the ABclonal mRNA-seq Lib Prep Kit (ABclonal, Wuhan, China). mRNA was purified from 1 μg of total RNA using oligo (dT) magnetic beads and fragmented in ABclonal First Strand Synthesis Reaction Buffer. The first strand of cDNA was synthesized using random primers and reverse transcriptase (RNase H) with the fragmented mRNA used as templates. Then, the second strand of cDNA was synthesized using DNA polymerase I, RNase H, buffer, and dNTPs. The synthesized double-stranded cDNA fragments were ligated with adaptor sequences for PCR amplification. The PCR products were purified, and the quality of the library was assessed using an Agilent Bioanalyzer 4150. Finally, sequencing was performed on the Illumina Novaseq 6000 platform.

Metabolites Extraction: After slowly the black soldier fly larva samples were slowly thawed at 4 °C, an appropriate amount of the sample was added to a pre-chilled solution of methanol/acetonitrile/water (2:2:1, *v/v*). The mixture was vortexed and subjected to low-temperature ultrasound for 30 min, then allowed to stand at −20 °C for 10 min. The samples were then centrifuged at 14,000× *g* at 4 °C for 20 min, and the supernatant was collected and vacuum dried. For mass spectrometry analysis, 100 µL of acetonitrile/water solution (acetonitrile = 1:1, *v/v*) was added for reconstitution, followed by vortexing. The samples were centrifuged at 14,000× *g* at 4 °C for 15 min, and the supernatant was taken for analysis. Then, 100 μL of sample was transferred to an EP tube. After the addition of 400 μL of extract solution (acetonitrile/methanol = 1:1, containing isotopically labeled internal standard mixture), the samples were vortexed for 30 s, sonicated for 5 min in an ice water bath, and incubated for 1 h at −40 °C to precipitate proteins. Then, the sample was centrifuged at 12,000 rpm for 15 min at 4 °C. The resulting supernatant was transferred to a fresh glass vial for analysis. The quality control (QC) sample was prepared by mixing an equal aliquot of the supernatants from all of the samples.

LC-MS analyses were performed using an UHPLC system (Vanquish, Thermo Fisher Scientific, Waltham, MA, USA) with a UPLC BEH Amide column (2.1 mm × 100 mm, 1.7 μm) coupled to a Q Exactive HFX mass spectrometer (Orbitrap MS, Thermo, Waltham, MA, USA). The mobile phase consisted of 25 mmol/L ammonium acetate and 25 ammonia hydroxide in water (pH = 9.75) (A) and acetonitrile (B). The auto-sampler temperature was 4 °C, and the injection volume was 2 μL. The QE HFX mass spectrometer was used for its ability to acquire MS/MS spectra on information-dependent acquisition (IDA) mode in the control of the acquisition software (Xcalibur, Thermo, Waltham, MA, USA). In this mode, the acquisition software continuously evaluates the full scan MS spectrum. The ESI source conditions were set as follows: sheath gas flow rate as 30 Arb, Aux gas flow rate as 25 Arb, capillary temperature 350 °C, full MS resolution as 60000, MS/MS resolution as 7500, collision energy as 10/30/60 in NCE mode, spray voltage as 3.6 kV (positive) or −3.2 kV (negative), respectively.

### 4.2. Bioinformatics Analysis

The data generated from the Illumina Beijing Genomics Institute platform were used for bioinformatics analysis. All analyses were conducted by Shanghai Biozeron Biotechnology Co., Ltd. (Shanghai, China) The main software and parameters used are as follows: raw data in fastq format were first processed using perl scripts to remove adaptor sequences and filter out low-quality reads, as well as those with a N content greater than 5%, resulting in clean reads for further analysis. The clean reads were aligned to the reference genome using HISAT2 software (Version 2.2.1) (http://daehwankimlab.github.io/hisat2/) (accessed on 28 June 2023) to obtain mapped reads for subsequent analysis. Feature Counts (http://subread.sourceforge.net/) (accessed on 28 June 2023) were used to calculate the number of reads mapped to each gene, and the FPKM (Fragments Per Kilobase of transcript per Million mapped reads) value for each gene was calculated based on its length.

Differential expression analysis between groups was conducted using DESeq2 (http://bioconductor.org/packages/release/bioc/html/DESeq2.html) (accessed on 28 June 2023), with the default thresholds for differentially expressed genes set at |log2FC| > 1 and Padj < 0.05. GO (Gene Ontology) and KEGG (Kyoto Encyclopedia of Genes and Genomes) enrichment analyses were performed on the differentially expressed genes to explain the functional enrichment of these genes and clarify the differences between samples at the gene functional level. GO functional enrichment and KEGG pathway enrichment analyses were performed using the cluster Profiler R package.

### 4.3. Statistical Analysis

After sum-normalization, the processed data were analyzed by R package (ropls), where they were subjected to multivariate data analysis, including orthogonal partial least-squares discriminant analysis (OPLS-DA). The 7-fold cross-validation and response permutation testing were used to evaluate the robustness of the model. The variable importance in the projection (VIP) value of each variable in the OPLS-DA model was calculated to indicate its contribution to the classification. Student’s *t* test was applied to determine the significance of differences between two groups of independent samples. VIP > 1 and a *p* value < 0.05 were used to screen significantly changed metabolites. Pearson’s correlation analysis was performed to determine the correlation between two variables.

## 5. Conclusions

This study provides strong evidence that substrate type significantly influences the physiological responses of BSFL. Our results show that BSFL fed with swill exhibits enhanced energy metabolism and growth, suggesting that swill is an optimal substrate for maximizing BSFL biomass production. In contrast, pig manure induces a stronger stress response, highlighting the importance of careful substrate selection in waste conversion processes. These findings not only deepen our understanding of the ecological role of BSFL in organic waste management but also provide clear guidance for optimal substrate selection in industrial applications. Specifically, swill, with its diverse nutritional composition, promotes growth and metabolism in BSFL, making it a valuable substrate for improving waste conversion efficiency in large-scale waste treatment systems. On the other hand, although pig manure is rich in nitrogen and minerals, its stress-inducing effects may limit the metabolic efficiency of BSFL. Therefore, selecting the right substrate based on its properties is crucial to maximizing waste conversion performance. This study provides essential molecular and physiological insights for improving the efficiency of BSFL in waste management. Future research could focus on further optimizing substrates and enhancing BSFL performance, thereby advancing their application in waste reduction, resource recycling, and sustainable production.

## Figures and Tables

**Table 1:** Content of nutrients in swill and pig manure matrix.

Group	Moisture Content	Organic Matter	Crude Protein	Crude Fat	Crude Ash	Calcium	Phosphorus
Swill	81.09 ± 2.97	62.58 ± 3.87	21.42 ± 1.51	34.84 ± 3.66	8.66 ± 1.54	1.95 ± 0.82	0.13 ± 0.02
Pig Manure	77.98 ± 2.17	16.64 ± 2.76	13.76 ± 2.38	2.84 ± 0.71	8.32 ± 2.13	0.81 ± 0.16	0.11 ± 0.04

**Table 2:** Changes in body weight of black soldier fly larvae at different stages (mg).

Group	Day 0 of the Experiment	Day 4 of the Experiment	Day 8 of the Experiment	Day 12 of the Experiment	Day 16 of the Experiment
Swill	30.72 ± 6.39	115.84 ± 18.42	196.86 ± 32.85	/	/
Pig Manure	32.26 ± 6.19	98.76 ± 15.72	181.93 ± 19.85	183.80 ± 20.29	181.54 ± 27.50

## Data Availability

The datasets presented in this study can be found in online repositories. The names of the repository and accession number(s) can be found in the NCBI SRA database with accession numbers PRJNA1159383 (http://www.ncbi.nlm.nih.gov/bioproject/1159383) (accessed on 9 September 2024).

## Supplementary Materials

The following supporting information can be downloaded at https://www.mdpi.com/article/10.3390/ijms252212147/s1.
